# Multifunctional Coating to Simultaneously Encapsulate Drug and Prevent Infection of Radiopaque Agent

**DOI:** 10.3390/ijms20092055

**Published:** 2019-04-26

**Authors:** Jiaying Li, Huan Wang, Qianping Guo, Caihong Zhu, Xuesong Zhu, Fengxuan Han, Huilin Yang, Bin Li

**Affiliations:** 1 College of Chemistry, Chemical Engineering and Materials Science, Orthopaedic Institute, Soochow University, Suzhou 215000, Jiangsu, China; lijiaying_1014@163.com (J.L.); hwang9520@stu.suda.edu.cn (H.W.); guoqianping@suda.edu.cn (Q.G.); zhucaihong@suda.edu.cn (C.Z.); suzhouspine@163.com (H.Y.); 2 China Orthopaedic Regenerative Medicine Group (CORMed), Hangzhou 310000, Zhejiang, China

**Keywords:** multifunctional coating, radiopaque agents, antibacterial activity, drug delivery

## Abstract

Poly(methyl methacrylate) (PMMA) bone cements have been widely used in clinical practices. In order to enhance PMMA’s imaging performance to facilitate surgical procedures, a supplementation of radiopaque agent is needed. However, PMMA bone cements are still facing problems of loosening and bacterial infection. In this study, a multifunctional coating to simultaneously encapsulate drug and prevent the infection of radiopaque agent has been developed. Barium sulfate (BaSO_4_), a common radiopaque agent, is used as a substrate material. We successfully fabricated porous BaSO_4_ microparticles, then modified with hexakis-(6-iodo-6-deoxy)-alpha-cyclodextrin (I-CD) and silver (Ag) to obtain porous BaSO_4_@PDA/I-CD/Ag microparticles. The porous nature and presence of PDA coating and I-CD on the surface of microparticles result in efficient loading and release of drugs such as protein. Meanwhile, the radiopacity of BaSO_4_@PDA/I-CD/Ag microparticles is enhanced by this multifunctional coating containing Ba, I and Ag. PMMA bone cements containing BaSO_4_@PDA/I-CD/Ag microparticles show 99% antibacterial rate against both *Staphylococcus aureus* (*S. aureus*) and *Escherichia Coli* (*E. coli*), yet without apparently affecting its biocompatibility. Together, this multifunctional coating possessing enhanced radiopacity, controlled drug delivery capability and exceptional antibacterial performance, may be a new way to modify radiopaque agents for bone cements.

## 1. Introduction

Poly(methyl methacrylate) (PMMA)-based bone cements have been widely used in orthopaedic open surgeries such as artificial joint replacement and minimally invasive surgeries such as vertebroplasty and kyphoplasty for treating vertebral fractures [1,2,3]. Although PMMA has excellent mechanical properties, it is not radiopaque. Therefore, a radiopaque agent is commonly needed in PMMA bone cements. Various radiopaque agents, including barium sulfate (BaSO_4_), zirconium dioxide (ZrO_2_), tungsten, tantalum, and iodinated methacrylate, have been used in PMMA bone cements [4,5,6,7,8,9,10]. Because of its excellent stability and outstanding optical properties, BaSO_4_ has been most commonly used as radiopaque agent in PMMA bone cements [9,11,12,13].

However, BaSO_4_ exists some problems in clinical application and needs to be further improved. As an inorganic filler, the chemical inertness nature of BaSO_4_ causes insufficient interaction between BaSO_4_ particles and PMMA matrix, adversely affecting the mechanical properties of bone cements [14]. In addition, BaSO_4_ has inherent cytotoxicity, which may lead to the loosening of implants [15]. Further modification of BaSO_4_ may improve its performance in PMMA bone cements. Micro/nano-sized BaSO_4_ particles have high specific surface area, and which may be benefit for improving its biocompatibility and optical imaging property [16]. In recent years, porous materials have been widely used in the field of catalysis, adsorption, and drug delivery due to their large specific surface area, tailored pore size, structure and surface properties [17]. With the purpose to further increase the specific surface area of BaSO_4_ particles to enhance biocompatibility and realize multifunction, porous BaSO_4_ particles are raised [11,16]. Nandakumar et al. prepared monodispersed spheroid BaSO_4_ microparticles with porous structure via direct precipitation of barium chloride (BaCl_2_) and ammonium sulfate ((NH_4_)_2_SO_4_) in aqueous polyvinyl alcohol (PVA) solution [11]. Chen et al. fabricated mesoporous BaSO_4_ microspheres with a larger pore size via Ostwald Ripening at room temperature, which will act as promising candidates for catalyst carrier, adsorbents, and so on [16].

A PDA-assisted modification method is appropriate for further modification of porous BaSO_4_ particles. The development of multifunctional radiopaque agent has become a trend. Hence, except X-ray absorption ability, the new radiopaque agent is expected to have antibacterial activity and therapeutic effect. Previous studies reported many surface modification techniques, among them PDA-assisted modification method is popular [18,19]. Dopamine molecules can undergo self-polymerization and form extraordinary adhesion on a wide variety of substrate surfaces under weak alkaline conditions [20]. More importantly, PDA coating can act as anchors to graft the secondary functional biopolymers with thiols and amines via Michael addition or Schiff base reactions. In the previous study, PDA was coated on to the surface of polystyrene culture plates for further decorated with liposomes [20]. Moreover, the catechol groups in PDA coating can also reduce metal ions to elemental metals, generating metallic microparticles on the surface of various materials.

The antibacterial property of BaSO_4_ particles could be realized by PDA-assisted Ag deposition. Ag modification is a useful method to inhibit bacterial infection [17,21,22,23]. Inflammation caused by bacteria is common in patients after surgeries. An important type of antibacterial material, Ag may help improve the antibacterial property of implants and facilitate healing when it is incorporated in the implants. Ag nanoparticles could be introduced to the surface of materials via reduction of Tollens’ reagent by PDA. In a previous study, we successfully modified poly(ether ether ketone) (PEEK) implants using PDA-based surface modification technology and subsequent deposition of Ag nanoparticles on the surface of the implants. This coating exhibited outstanding antibacterial property against both *Staphylococcus aureus* (*S. aureus*) and *Escherichia Coli* (*E. coli*) in vitro as well as decent antibacterial performance in vivo [24]. Therefore, the technique of PDA-assisted deposition of Ag may be also suitable for BaSO_4_ particles.

The therapeutic effect of BaSO_4_ particles as radiopaque agent could be endowed with loading therapeutic drugs by porous structure, PDA coating and cyclodextrin (CD). The porous BaSO_4_ particles can load drugs [17]. PDA modification may also promote protein adsorption on the surface of particles [20]. There are different methods to encapsulate drugs, among them I-CD represents excellent drug loading property contributing to inclusion complex formation of CD with a wide array of small molecules [25,26,27,28]. Rivera-Delgado et al. synthesized different cyclodextrin polymers to control the delivery rates from solid polymer from hours and days, to weeks and months [28]. In addition, CD can also help to inhibit the aggregation of therapeutic proteins released from the porous particles. Because the CD can incorporate hydrophobic residues on aggregation-prone proteins or on their partially unfolded intermediates into the hydrophobic cavity [29]. Therefore, I-CD will be introduced into the PDA coating for drug encapsulation. The presence of porous structure, PDA coating and CD could control the delivery and maintain the bioactive of therapeutic protein drugs. In addition, the presence of I may also enhance the X-ray absorption ability.

**Scheme 1:**
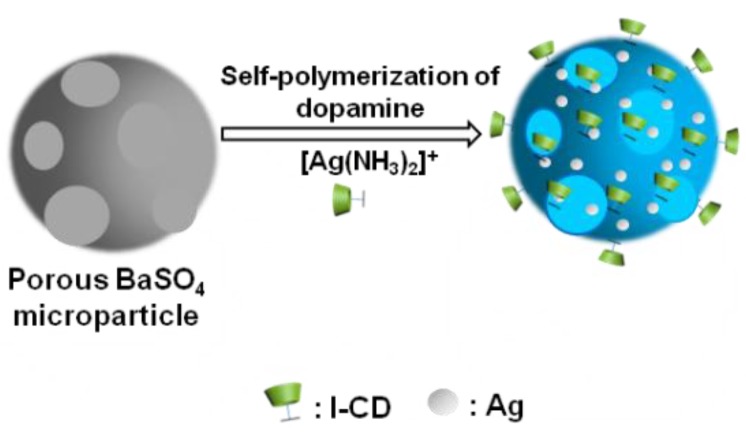
Schematic of the preparation of multifunctional porous BaSO_4_@PDA/I-CD/Ag microparticle.

With the goal to gain a radiopaque agent having strong radiopacity, great antibacterial property and long drug delivery ability, we developed novel porous BaSO_4_@PDA/I-CD/Ag microparticles with multifunctional coating (Scheme 1). As both BaSO_4_ and iodo-compounds are common radiopaque agents in radiography and computed tomography (CT), the combination of them may further improve the radiopacity of radiopaque agent [30]. The PDA coating, polymerized from dopamine in alkaline condition, has been proved to improve the biocompatibility as surface coating outside the microparticles. The PDA coating, I-CD and pores in BaSO_4_ microparticles can load drugs for promoting bone formation. Moreover, in situ deposition of Ag on the surface of the PDA coating could be realized, that will endow the material with an antibacterial property. Scanning electron microscope (SEM) and energy-dispersive X-ray spectroscope (EDS) will be used to observe the structure and measure elementary composition of porous BaSO_4_@PDA/I-CD/Ag microparticles. The drug release behavior of porous BaSO_4_@PDA/I-CD/Ag microparticles is going to be detected. X-ray absorption measurements and mechanical property of PMMA bone cements containing porous BaSO_4_@PDA/I-CD/Ag microparticles will also be evaluated. The mechanical property of PMMA bone cements containing particles will be studied. The antibacterial performance of PMMA bone cements added porous BaSO_4_@PDA/I-CD/Ag microparticles will be investigated using *S. aureus* and *E. coli*. The biocompatibility of PMMA bone cement containing porous BaSO_4_@PDA/I-CD/Ag microparticles will be determined using MC3T3-E1 cells.

## 2. Results

### 2.1. Characterization of Microparticles

**Figure 1:**
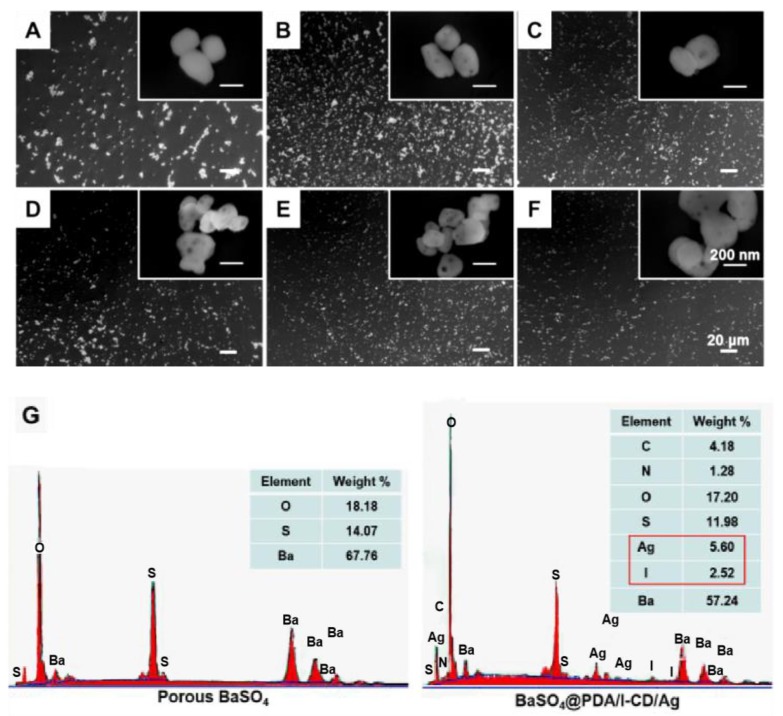
SEM images of (**A**) BaSO_4_; (**B**) porous BaSO_4_; (**C**) porous BaSO_4_@PDA; (**D**) porous BaSO_4_@PDA/I-CD, (**E**) porous BaSO_4_@PDA/Ag and (**F**) porous BaSO_4_@PDA/I-CD/Ag microparticles. (**G**) EDS spectra of porous BaSO_4_ and porous BaSO_4_@PDA/I-CD/Ag microparticles.

The morphology of prepared microparticles is characterized by SEM and TEM. To prepare porous BaSO_4_ microparticles, BaSO_4_ microparticles are firstly prepared in the presence of PVA. The diameter of obtained BaSO_4_ microparticles is in the range of 50–200 nm (Figure S1). Porous BaSO_4_ microparticle is then prepared by calcining the BaSO_4_ microparticles at 600 °C. From the SEM image, it is observed that the diameter of BaSO_4_ microparticle would not change (Figure S1). TEM images showed that PDA coating was successfully generated on the surface of porous BaSO_4_ (Figure S2B–D). Moreover, obvious pores appear in porous BaSO_4_ microparticles (Figure 1 and Figure S2). In addition, both the diameter and pore structure doesn’t change obviously after PDA coating or other further modification to prepare porous BaSO_4_@PDA, BaSO_4_@PDA/Ag, BaSO_4_@PDA/I-CD and BaSO_4_@PDA/I-CD/Ag particles (Figure 1C–F, Figure S2B–D). To investigate whether Ag and I elements have been successfully added in BaSO_4_@PDA/I-CD/Ag microparticle, EDS spectra of BaSO_4_ and BaSO_4_@PDA/I-CD/Ag is shown in Figure 1G. The results show that both Ag and I elements are present in BaSO_4_@PDA/I-CD/Ag particles. Moreover, the content of Ag and I elements in porous BaSO_4_@PDA/I-CD/Ag microparticles are about 5.60 wt% and 2.52 wt%, respectively.

### 2.2. In Vitro Drug Loading and Release Behavior of Microparticles with Multifunctional Coating

**Figure 2:**
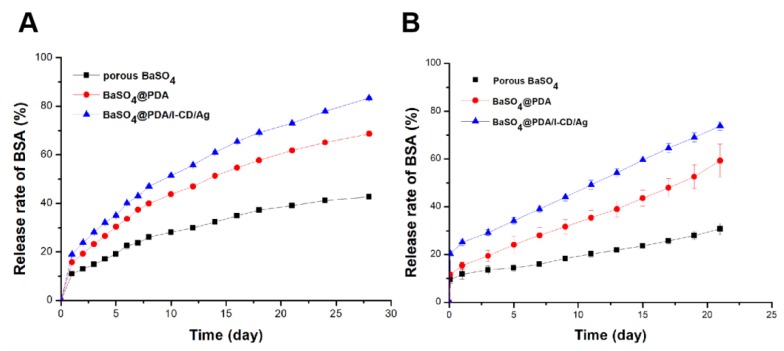
In vitro release of BSA from porous BaSO_4_, BaSO_4_@PDA, BaSO_4_@PDA/I-CD/Ag particles (**A**) and PMMA bone cements containing porous BaSO_4_, BaSO_4_@PDA, BaSO_4_@PDA/I-CD/Ag particles (**B**).

The drug loading behavior of porous BaSO_4_, porous BaSO_4_@PDA and porous BaSO_4_@PDA/I-CD/Ag particles are detected in this experiment. BSA is chosen as a model protein drug in this test. All the particles can load and sustained release BSA (Figure 2A). The loading efficiency of bovine serum albumin (BSA) in porous BaSO_4_, BaSO_4_@PDA and BaSO_4_@PDA/I-CD/Ag are about 67.5%, 82.6% and 85.4%, respectively (Figure S3). Compared to porous BaSO_4_, porous BaSO_4_@PDA shows higher encapsulation rate. The introduction of I-CD on the surface of BaSO_4_ microparticles is proved to be effective for drug loading, because higher loading efficiency is observed in BaSO_4_@PDA/I-CD/Ag than BaSO_4_@PDA/I-CD microparticle.

The drug delivery behaviors of porous BaSO_4_ microparticles after modifying by multifunctional coating are shown in Figure 2A. The burst release of porous BaSO_4_ is about 10%, while it increases to about 20% for porous BaSO_4_@PDA/I-CD/Ag microparticles. A faster release rate is also observed in BaSO_4_@PDA and BaSO_4_@PDA/I-CD/Ag particles. The measured release rate of porous BaSO_4_@PDA and porous BaSO_4_@PDA/I-CD/Ag are relatively close. However, the cumulative amounts of BSA in three particles are also different. After 28 d, the cumulative release of BSA in porous BaSO_4_@PDA/I-CD/Ag reaches 83.4%, higher than porous BaSO_4_ (42.6%) incubated in 37 °C.

To further investigate the drug release property of microparticle, we added BSA-loaded particle into PMMA bone cements. As shown in Figure 2B, the release rate of PMMA bone cements containing porous BaSO_4_, BaSO_4_@PDA and BaSO_4_@PDA/I-CD/Ag particles were inferior to the pure particles at the same time point, respectively. Additionally, the cumulative release amount of BSA from PMMA bone cement containing BaSO_4_@PDA/I-CD/Ag particles is higher than the other samples. After 21 d, the release rate of BSA from PMMA bone cements containing BaSO_4_@PDA/I-CD/Ag particles reach up to ~73% higher than PMMA bone cements containing BaSO_4_@PDA (59%) and porous BaSO_4_ (30%) particles

### 2.3. Effect of Multifunctional Coating on Radiopacity of Microparticles

**Figure 3:**
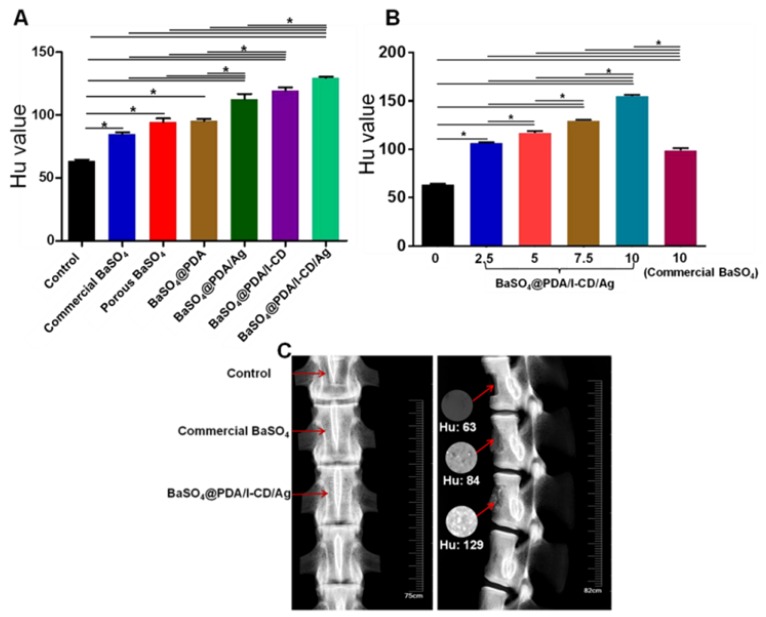
(**A**) Hu values by micro-CT of PMMA bone cements containing 7.5 wt% commercial BaSO_4_, porous BaSO_4_, porous BaSO_4_@PDA, porous BaSO_4_@PDA/Ag, porous BaSO_4_@PDA/I-CD and porous BaSO_4_@PDA/I-CD/Ag microparticles. (**B**) Hu values of bone cements containing 0, 2.5 wt%, 5 wt%, 7.5 wt%, 10 wt% porous BaSO_4_@PDA/I-CD/Ag microparticles and 10 wt% commercial BaSO_4_ particles. (**C**) X-ray absorption property of the defect region in isolated vertebral body of sheep filled with PMMA bone cement (Control) and PMMA bone cements containing 7.5 wt% commercial BaSO_4_ microparticles (Commercial BaSO_4_) and porous BaSO_4_@PDA/I-CD/Ag microparticles (BaSO_4_@PDA/I-CD/Ag).* *p* < 0.05.

The radiopacity of PMMA bone cements with various microparticles is detected by micro-CT scanning. Figure 3A shows the Hu values of PMMA bone cement (Control) and PMMA bone cements containing 7.5 wt% commercial BaSO_4_, porous BaSO_4_, BaSO_4_@PDA, BaSO_4_@PDA/Ag, BaSO_4_@PDA/I-CD and BaSO_4_@PDA/I-CD/Ag microparticles. The Hu value of pure PMMA bone cement is 63.6 ± 1.44. After adding 7.5 wt% commercial BaSO_4_, the Hu value of PMMA bone cement increases to 84.9 ± 1.87. The Hu values of PMMA bone cements containing 7.5 wt% porous BaSO_4_ and BaSO_4_@PDA are 94.4 ± 4.42 and 95.4 ± 2.12, respectively. However, there is no significant difference among PMMA bone cements containing commercial BaSO_4_, porous BaSO_4_ and BaSO_4_@PDA microparticles. The Hu values of BaSO_4_@PDA/Ag, BaSO_4_@PDA/I-CD and BaSO_4_@PDA/I-CD/Ag are 112.7 ± 5.75, 119.4 ± 3.60 and 129.6 ± 1.36, respectively.

The adding amount of BaSO_4_@PDA/I-CD/Ag microparticles in the PMMA bone cements also affected the radiopacity. The Hu values of PMMA bone cements containing 0, 2.5 wt%, 5 wt%, 7.5 wt% and 10 wt% BaSO_4_@PDA/I-CD/Ag microparticles are 63.6 ± 1.44, 106.6 ± 1.11, 117.1 ± 2.60,129.6 ± 1.36 and 155.1 ± 1.74, respectively (Figure 3B). The result shows that the Hu value of PMMA bone cements increases with the amount of porous BaSO_4_@PDA/I-CD/Ag microparticles improved. However, the Hu value of PMMA bone cement containing 7.5 wt% porous BaSO_4_@PDA/I-CD/Ag microparticles is still higher than PMMA bone cement containing 10% commercial BaSO_4_.

The X-ray absorption property of PMMA bone cement and PMMA bone cements containing 7.5 wt% commercial BaSO_4_ microparticles and 7.5 wt% porous BaSO_4_@PDA/I-CD/Ag microparticles, injected in defect of isolated vertebral body of sheep, is detected. The Hu value of PMMA bone cement is 63, while the Hu value increases to 84 and 129 after adding commercial BaSO_4_ particles and BaSO_4_@PDA/I-CD/Ag microparticles, respectively (Figure 3C).

### 2.4. The Mechanical Property of Bone Cements Containing Microparticles with Multifunctional Coating

The morphology and diameter distribution of PMMA particles for preparing PMMA bone cements in this study are provided in Figure S4. As shown, the PMMA particles we bought from company are spherical. The diameter of these particles is in the range of 100–250 μm. It is well known that the size of PMMA particles will affect the mechanical property of PMMA bone cements. To eliminate this error, the same PMMA particles are used in the following mechanical tests.

The effects of particles on the mechanical property of PMMA bone cements are evaluated in this study. The compressive strength of pure PMMA bone cements prepared using this PMMA particles is 97.2 ± 0.03 MPa (Figure S5A). The compressive strength of PMMA bone cements containing 2.5 wt%, 5 wt%, 7.5 wt% and 10 wt% porous BaSO_4_@PDA/I-CD/Ag microparticles are 94.0 ± 0.08 MPa, 90.0 ± 0.03 MPa, 86.6 ± 0.22 MPa and 85.3 ± 0.06 MPa, respectively. The results show that the compressive strength of PMMA bone cements significantly decreases with increasing amount of BaSO_4_@PDA/I-CD/Ag microparticles. The effect of different particles on the mechanical property of PMMA bone cements is also determined. The compressive strength of PMMA bone cements containing 7.5 wt% of commercial BaSO_4_, porous BaSO_4_, BaSO_4_@PDA, BaSO_4_@PDA/Ag, BaSO_4_@PDA/I-CD and BaSO_4_@PDA/I-CD/Ag microparticles are 87.6 ± 0.04 MPa, 87.9 ± 0.15 MPa, 87.1 ± 0.08 MPa, 86.8 ± 0.04 MPa, 86.1 ± 0.07 MPa and 86.6 ± 0.22 MPa, respectively (Figure S5B).

Male sheep lumbar vertebrae (L1–L6) was used to investigate the mechanical property of PMMA bone cement containing 7.5 wt% of porous BaSO_4_, and BaSO_4_@PDA/I-CD/Ag microparticles. As shown in Figure S6, compared with commercial BaSO_4_, BaSO_4_@PDA/I-CD/Ag particles added PMMA bone cement could improve Young’s modulus of lumbar vertebrae significantly. The Young’s modulus of lumbar vertebrae with defect is 0.54 ± 0.057 GPa. The Young’s modulus of lumbar vertebrae filled with PMMA bone cement containing BaSO_4_@PDA/I-CD/Ag particles reaches 0.89 ± 0.060 GPa, higher than lumbar vertebrae filled with PMMA bone cements containing commercial BaSO_4_ (0.83 ± 0.064 GPa), indicating that BaSO_4_@PDA/I-CD/Ag particles contained PMMA bone cement could enhance the mechanical property of injured vertebrae.

### 2.5. The Antibacterial Property of Bone Cements Containing Microparticles with Multifunctional Coating

**Figure 4:**
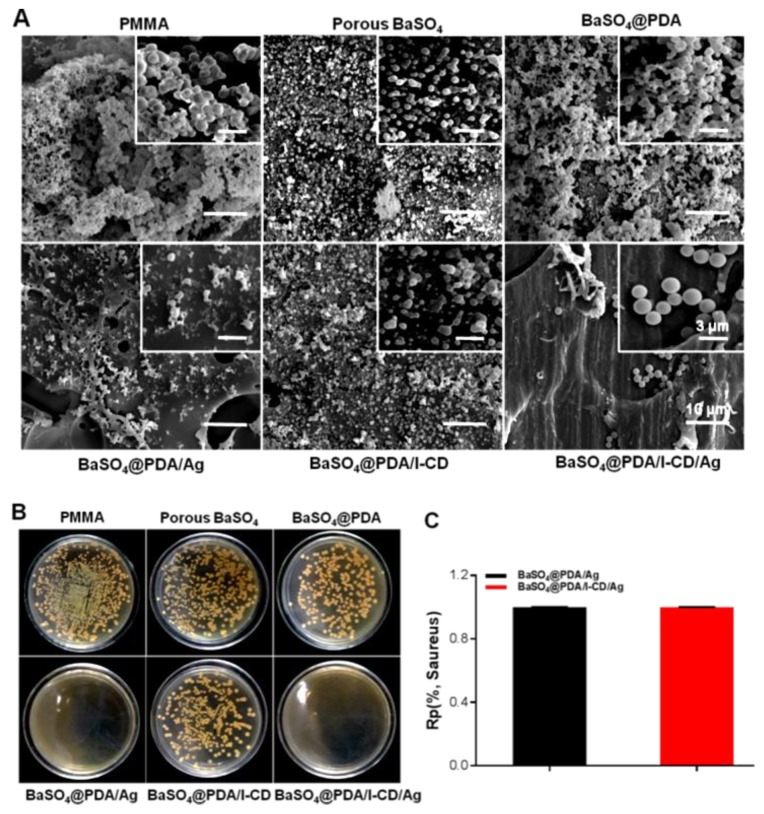
(**A**) SEM images of PMMA bone cement and PMMA bone cements containing 7.5 wt% different particles after culture of *S. aureus* for 24 h. (**B**) Colonies of *S. aureus* on different samples were counted. (**C**) Inhibitory rate of PMMA bone cements containing 7.5 wt% porous BaSO_4_@PDA/Ag or BaSO_4_@PDA/I-CD/Ag microparticles compared to PMMA bone cements against *S. aureus*.

**Figure 5:**
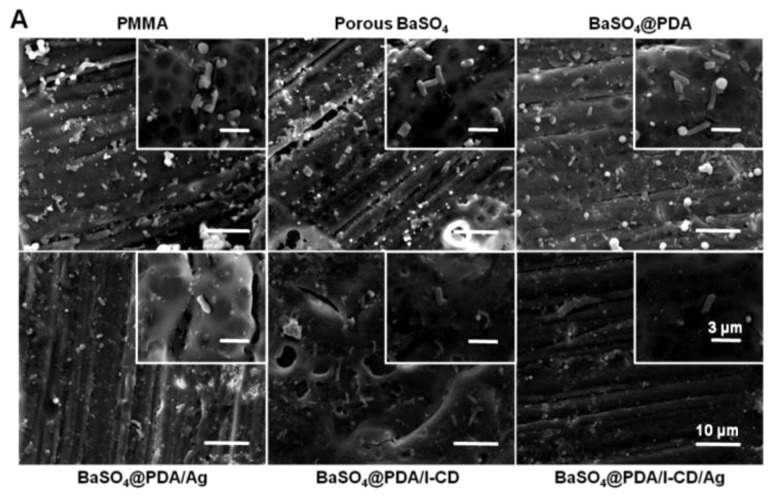

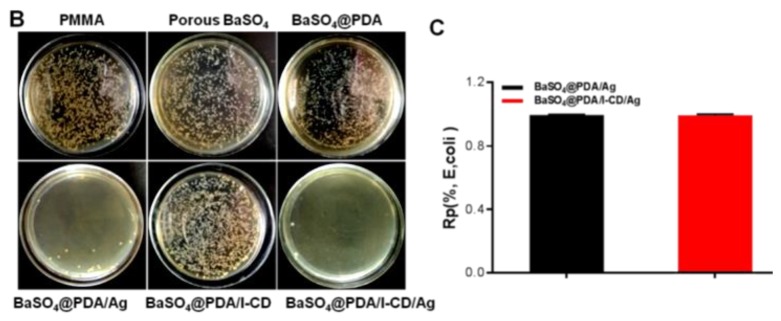
(**A**) SEM images of PMMA bone cement and PMMA bone cements containing 7.5 wt% different particles after culture of *E. coli* for 24 h. (**B**) Colonies of *E. coli* on different samples were counted. (**C**) Inhibitory rate of PMMA bone cements containing 7.5 wt% porous BaSO_4_@PDA/Ag or BaSO_4_@PDA/I-CD/Ag microparticles compared to PMMA bone cements against *E. coli*.

The antibacterial property of PMMA bone cement and PMMA bone cements containing 7.5 wt% porous BaSO_4_@PDA/Ag and porous BaSO_4_@PDA/I-CD/Ag microparticles are evaluated using both *S. aureus* (Gram-positive bacteria) and *E. coli* (Gram-negative bacteria) which are the most common pathogens causing implant infection in clinic. The bacteria seeded on the surface of PMMA bone cements is observed via SEM after cultured 24 h. As shown in Figure 4A, the surface of PMMA bone cements is teeming with a lot of *S. aureus*. There are also many *S. aureus* on the surface of PMMA bone cements added porous BaSO_4_, porous BaSO_4_@PDA and porous BaSO_4_@PDA/I-CD microparticles. However, only a small amount of bacteria is observed on the surface of PMMA bone cements containing porous BaSO_4_@PDA/Ag and porous BaSO_4_@PDA/I-CD/Ag microparticles. Compared to pure PMMA bone cement, the inhibitory rate of PMMA bone cements containing porous BaSO_4_@PDA/Ag or porous BaSO_4_@PDA/I-CD/Ag microparticles against *S. aureus* reaches above 99% (Figure 4B,C). Figure 5 shows that the growth of *E. coli* is also inhibited in the presence of Ag. Both the inhibitory rates of PMMA bone cements containing porous BaSO_4_@PDA/Ag or BaSO_4_@PDA/I-CD/Ag microparticles achieve 99% against to *E. coli*.

**Figure 6:**
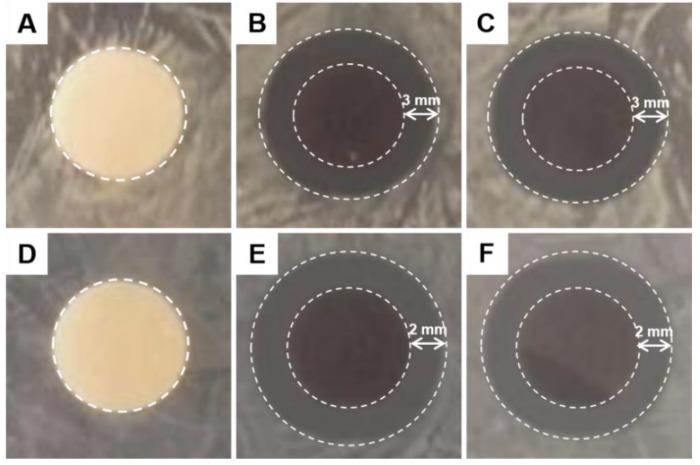
Inhibition zones of PMMA bone cements containing commercial BaSO_4_ (**A,D**), BaSO_4_@PDA/Ag (**B,E**) and BaSO_4_@PDA/I-CD/Ag (**C,F**) contact with *S. aureus* (**A**–**C**) and *E. coli* (**D**–**F**) at 37 °C after 24 h.

As shown in Figure 6, no inhibition zone is observed in PMMA bone cement containing commercial BaSO_4_ (Figure 6A,D). However, the PMMA bone cements containing BaSO_4_@PDA/Ag (Figure 6B,E) and BaSO_4_@PDA/I-CD/Ag particles (Figure 6C,F) show distinct zone of inhibition against *S. aureus* and *E. coli*. The radius of inhibition zone for PMMA bone cements containing BaSO_4_@PDA/Ag and BaSO_4_@PDA/I-CD/Ag particles is about 3 mm (Figure 6B,C). And the radius of inhibition zone for PMMA bone cements containing BaSO_4_@PDA/Ag and BaSO_4_@PDA/I-CD/Ag particles is about 2 mm (Figure 6E,F). The results demonstrate the excellent inhibition properties of BaSO_4_@PDA/Ag and BaSO_4_@PDA/I-CD/Ag particles, indicating the functional coating containing silver on the surface of microparticles having excellent antibacterial activity.

### 2.6. In Vitro Biocompatibility of Bone Cements Containing Microparticles with Multifunctional Coating

**Figure 7:**
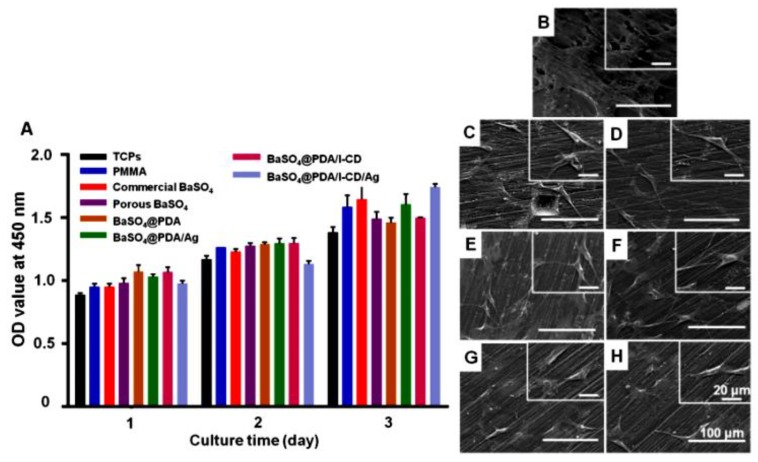
(**A**) Cytotoxicity analysis of MC3T3-E1 cells cultured with extract liquid of PMMA bone cement and PMMA bone cements containing 7.5 wt% different particles. (**B-H**) MC3T3-E1 cells morphology on the surface of PMMA bone cement (**B**) and PMMA bone cements containing 7.5 wt% commercial BaSO_4_ (**C**), porous BaSO_4_ (**D**), BaSO_4_@PDA (**E**), BaSO_4_@PDA/Ag (**F**), BaSO_4_@PDA/I-CD (**G**) and BaSO_4_@PDA/I-CD/Ag (**H**).

To test cytotoxicity, MC3T3-E1 cells are cultured with various extract liquids from PMMA bone cement and PMMA bone cements added commercial BaSO_4_, porous BaSO_4_, porous BaSO_4_@PDA, porous BaSO_4_@PDA/Ag, porous BaSO_4_@PDA/I-CD and porous BaSO_4_@PDA/I-CD/Ag particles (7.5 wt%). The proliferation of MC3T3-E1 cells at 1, 2 and 3 days is evaluated via Cell Counting Kit-8 (CCK-8). As shown in Figure 7A, OD values of different groups all increase with culture time. The morphology of MC3T3-E1 cells on PMMA bone cements containing various particles is detected (Figure 7B–H). For porous BaSO_4_@PDA/I-CD/Ag group, MC3T3-E1 cells show favorable cell adhesion and spread on the surface of PMMA bone cements as well as pure PMMA group. The cell adhesion and morphology among different groups have no difference. Compared with the PMMA group, the cells on bone cements containing porous BaSO_4_@PDA/I-CD/Ag also grow well, and the cells are more spread.

## 3. Discussion

The aim of this study is to prepare multifunctional radiopaque agent for bone cements. BaSO_4_ microparticle, a common radiopaque agent, is used as a substrate material for further modification. According to the SEM results, it can be seen that porous BaSO_4_ microparticle has been successfully fabricated. The pores in the particles can make porous BaSO_4_ microparticles itself became a good carrier for therapeutic drugs. Similarly, the pores may also be used to increase drug loading for other radiopaque agent. To realize the multifunction, further surface modification of this porous BaSO_4_ microparticle is carried out. In this study, PDA coating is used as a platform for modification, which is an easy method for surface modification, is not only applied to modify most materials but also forms functional groups for further reaction [17,20,21,22]. EDS result shows that Ag and I-CD are modified on the surface of porous BaSO_4_ microparticles with the help of PDA coating. Porous BaSO_4_ microparticles may absorb some Ag and I-CD to a certain extent because of existed pores, but the PDA coating will improve the stability and amount of Ag and I-CD. Additionally, the PDA coating may also improve the interface strength between PMMA and BaSO_4_ particles and be used to absorb drugs.

Because of the porous structure, BSA can be encapsulated into porous BaSO_4_ microparticle. Compared to porous BaSO_4_, porous BaSO_4_@PDA shows higher encapsulation rate. This result may be attributed to protein adsorption of PDA coating. It is well known that CD can load drugs contributing to inclusion complex formation of CD with a wide array of small molecules and stabilize protein bioactivity in the liquid and dried state by inhibiting the protein aggregation [25,26,27,28,29], combining the function of PDA adsorption and CD, porous BaSO_4_@PDA/I-CD/Ag microparticle exhibits highest drug loading efficiency. Compared with porous BaSO_4_, porous BaSO_4_@PDA, porous BaSO_4_@PDA/I-CD/Ag microparticles exhibited burst release of BSA. The faster release rate of pure particles and cements containing porous BaSO_4_@PDA and porous BaSO_4_@PDA/I-CD/Ag microparticles than porous BaSO_4_ microparticles may be attributed to the improvement of hydrophilicity after PDA coating. Although the release rate of BaSO_4_@PDA/I-CD/Ag is faster, the released amount of BSA in this group at each time point is most. The results show that the designed multifunctional coatings on the surface of porous BaSO4 microparticles work as intended. Therefore, the prepared porous BaSO_4_@PDA/I-CD/Ag microparticles still have potential application as a drug carrier.

PMMA bone cement shows excellent mechanical property and its drug loading and release property is demand. Utilizing mesoporous silica nanoparticles to load antibiotics can improve anti-infection property of bone cement. The incorporation of MSNs to the bone cements (8.15 wt%) shows no detrimental effects on the biomechanical properties of bone cements, meanwhile sustained drug delivery from bone cement endows cement anti-infection property and promotes its therapeutic. CD can load various drugs, the addition of CD microparticles to cement enables incorporation of previously incompatible antibiotics while preserves favorable mechanical properties [23,25,31]. In this study, the fabricated porous BaSO_4_@PDA/I-CD/Ag microparticle in PMMA bone cement can load and release protein. Compared with cement containing porous BaSO_4_, samples containing porous BaSO_4_@PDA/I-CD/Ag microparticle can release more BSA at the same time due to the multifunctional coating. Therefore, PMMA bone cement containing porous BaSO_4_@PDA/I-CD/Ag microparticle may be a potential drug carrier.

PMMA bone cement containing porous BaSO_4_@PDA/I-CD/Ag microparticles showed good X-ray imaging efficiency. I element has good X-ray absorption property and it is often used as contrast agents in clinical practice. Ag, as a metallic element, can also absorb X-ray. Here, the radiopacity of BaSO_4_@PDA/Ag and BaSO_4_@PDA/I-CD are significantly enhanced by the introduction of Ag or I. PMMA bone cements containing above microparticles exhibit excellent X-ray imaging property and porous BaSO_4_@PDA/I-CD/Ag may be applied as a new type radiocontrast for clinical therapeutics.

It was reported that the addition of additives into PMMA bone cements would decrease the mechanical property [32,33]. In our group’s previous study, the compressive strength also decreased with addition of magnesium balls [33]. This study shows the same results with previous reports. The results demonstrate that the addition of particles will reduce the mechanical properties of PMMA bone cements slightly. Even the amount of porous BaSO_4_@PDA/I-CD/Ag microparticles in bone cements reaches 10 wt%, the compressive strength is larger than standard (ISO 5833, 70 MPa). That is contributed to the multifunctional coating on the surface of porous BaSO_4_ particle, it can enhance the interaction between particles and PMMA bone cement matrix via functional groups of PDA coating. Therefore, PMMA bone cement can maintain a high compressive strength even if adding a large amount of porous BaSO_4_@PDA/I-CD/Ag. The mechanical property of lumbar vertebrae filled with cement containing BaSO_4_@PDA/I-CD/Ag microparticles is higher than samples containing commercial BaSO_4_ particles, further demonstrating that BaSO_4_@PDA/I-CD/Ag microparticles can improve the strength of cement.

Silver-based nanomaterials are known for their strong antibacterial activities for a lot of bacteria [17,21,22]. Previous studies indicated the presence of catechol structure in polydopamine could inhibit the growth and proliferation of bacteria in some extent. However, the antibacterial ability of PDA is far less than the actual clinical needs. In this study, silver is in situ deposited onto the surface of porous BaSO_4_@PDA microparticles with the help of PDA-based surface modification technology to gain porous BaSO_4_@PDA/Ag or porous BaSO_4_@PDA/I-CD/Ag microparticles. As shown, even the additional amount of BaSO_4_@PDA/Ag or porous BaSO_4_@PDA/I-CD/Ag microparticles is only 7.5 wt%, remarkable antibacterial effect is obtained. The results show that the multifunctional coating on the surface of microparticles is effective in preventing bacterial infection. Therefore, this multifunctional coating may be favorable for radiopaque agent modification.

The biocompatibility of radiopaque agents is another factor affecting the clinical application of bone cements. In this study, 7.5 wt% is thought to be an optimized amount. Then the cytotoxicity of the extracts of PMMA bone cement and PMMA bone cements adding commercial BaSO_4_, porous BaSO_4_, porous BaSO_4_@PDA, porous BaSO_4_@PDA/Ag and porous BaSO_4_@PDA/I-CD or porous BaSO_4_@PDA/I-CD/Ag particles are detected using MC3T3-E1 cells. PDA is a kind of functional polymer coating, which not only does not produce toxicity, but also improves the biocompatibility of substrate materials and promotes cell adhesion. Compared with pure PMMA bone cement, the samples adding different particles show no obvious cytotoxicity. Hence, this multifunctional coating may be a safe approach for radiopaque agent modification.

## 4. Materials and Methods

### 4.1. Preparation and Characterizations of Porous BaSO_4_@PDA/I-CD/Ag Microparticles

Porous BaSO_4_ microparticles were prepared according to the literature [11]. In brief, 5.2 g BaCl_2_ (Sinopharm Chemical Reagent Co., Ltd., Shanghai, China) was dissolved in 50 mL deionized water and thoroughly mixed with 50 mL 3 wt% PVA (Sinopharm Chemical Reagent Co., Ltd., Shanghai, China) solution under vigorous stirring. Then 25 mL deionized water containing 3.3 g (NH_4_)_2_SO_4_ (Sinopharm Chemical Reagent Co., Ltd., Shanghai, China) was dropped into the above solution through a constant pressure titration funnel at a rate of two drops per second. After that, the mixture was settled for 5 h at room temperature. The precipitate was thoroughly washed with deionized water to remove redundant PVA, unreacted reagents and by-products and then dried at 80 °C for 2 h to obtain BaSO_4_ microparticles. Porous BaSO_4_ microparticles were prepared by further calcining the BaSO_4_ microparticles at 600 °C for 4 h.

To prepare porous BaSO_4_@PDA microparticles, 3 g porous BaSO_4_ microparticles were dispersed in a dopamine (Sinopharm Chemical Reagent Co., Ltd., Shanghai, China) solution, which was prepared by dissolving 300 mg dopamine hydrochloride (Sinopharm Chemical Reagent Co., Ltd., Shanghai, China) and 120 mg copper sulfate (CuSO_4_) (Sinopharm Chemical Reagent Co., Ltd., Shanghai, China) in 150 mL Tris solution (50 mM, pH = 8.5). After 111 μL of hydrogen peroxide (H_2_O_2_) (Chengdu area of the ndustrial development zone xindu mulan, Chengdu, China) was added, the mixture was stirred for 40 min at 25 °C. Further, porous BaSO_4_@PDA/I-CD microparticles were prepared by adding 1 mL *N*, *N*-dimethylformamide (DMF) (Sinopharm Chemical Reagent Co., Ltd., Shanghai, China) solution containing 100 mg I-CD (Zhiyuan Biotechnology Co., Ltd., Binzhou, China) quickly prior to adding H_2_O_2_. To obtain porous BaSO_4_@PDA/Ag microparticles, 3 g porous BaSO_4_ microparticles were added into 150 mL Tollens’ solution (0.02 M) and then mixed with 150 mL dopamine solution (2 mg mL^−1^), followed by stirring at 25 °C for 8 h. Finally, the porous BaSO_4_@PDA/I-CD/Ag microparticles were fabricated by mixing 150 mL Tollens’ reagent (0.02 M) containing 3 g BaSO_4_ microparticles, and 150 mL dopamine solution (2 mg mL^−1^) containing 100 mg I-CD. This mixture was vigorously stirred at 25 °C for 8 h to obtain porous BaSO_4_@PDA/I-CD/Ag microparticles.

SEM (Quanta 250, FEI, Hillsboro, OR, USA) and TEM (Tecnai G2 F20, OR, USA) were used to characterize the morphology of all microparticles. EDS (INCA X-Max 250, EDAX Inc, PA, USA) was used for surface chemical analysis of microparticles.

### 4.2. Drug Release Measurements

In order to measure the drug delivery behavior of particles, BSA (Guangzhou Saiguo Biotech Co., Ltd., Guangzhou, China) was used a model drug in this study. In brief, 100 mg porous BaSO_4_, porous BaSO_4_@PDA or porous BaSO_4_@PDA/I-CD/Ag were dispersed in 1 mL BSA solution (2 mg mL^−1^), and shaken at 37 °C for 2 h. Drug-loaded microparticles were obtained by centrifuging and drying in vacuum oven overnight. For the drug release tests, 20 mg BSA-loaded microparticles were added into 1 mL phosphate buffer saline (PBS) (Hyclone, UT, USA) solution and shaken at 37 °C. At designed time points, 0.25 mL supernatant was sampled and 0.25 mL fresh PBS was added. Three duplicates were analyzed at each time point in this test. The concentration of BSA was analyzed by using a micro BCA protein assay kit (Beijing ComWin Biotech Co., Ltd., Beijing, China) at the wavelength of 562 nm. The release of BSA from PMMA bone cement containing different particles was also investigated and the method to fabricate PMMA bone cement was as followed.

### 4.3. Preparation of PMMA Bone Cements Containing Microparticles

PMMA bone cements contain powder and liquid, the mass to volume ratio of them is 2:1. The solid includes PMMA (M. W 35,000, Sigma-Aldrich, Shanghai, China), radiopaque agent and 2% (*w*/*w*) di-benzoyl peroxide (BPO) (Sigma-Aldrich, Shanghai, China). While the liquid is consisted of 99% methyl methacrylate (MMA) (Sinopharm Chemical Reagent Co., Ltd., Shanghai, China) and 1% (*v*/*v*) *N*,*N*-dimethyl-p-toluidine (DMPT) (Sinopharm Chemical Reagent Co., Ltd., Shanghai, China). Herein, 3 g powder and 1.5 mL liquid were used to fabricate pure PMMA bone cements. In this study, four types of mass ratio about the porous BaSO_4_@PDA/I-CD/Ag microparticles to solid phase (2.5 wt%, 5 wt%, 7.5 wt%, 10 wt%) were added in PMMA bone cements. The production of PMMA bone cements containing 2.5 wt% porous microparticles is described as an example. The solid powder including 2.925 g PMMA, 0.075 g porous microparticles and 0.006 g BPO were mixed in mortar, 1.5 mL well-mixed liquid including 1.485 mL MMA and 0.015 mL DMPT was added into the mortar. The mixture was removed to molds before solidification. PMMA bone cements containing porous microparticles could be obtained a few minutes later. The bone cements containing commercial BaSO_4_, porous BaSO_4_, porous BaSO_4_@PDA, porous BaSO_4_@PDA/Ag and porous BaSO_4_@PDA/I-CD microparticles were prepared with the same method mentioned above.

### 4.4. Radiopacity Measurements

The cylindrical PMMA bone cements (height: 12 mm, diameter: 6 mm) containing 2.5 wt%, 5 wt%, 7.5 wt% and 10 wt% porous BaSO_4_@PDA/I-CD/Ag microparticles were measured via micro-CT scanning (SkyScan 1176, SkyScan, Aartselaar, Belgium) for Hu value analysis. Three samples were detected for each group. The radiopacity of the PMMA bone cements formulated with different radiopaque agents in a defect (length: 20 mm, width: 20 mm, height: 10 mm) of isolated vertebral body of sheep was also detected. Before the X-ray imaging, PMMA bone cements (Control group), PMMA bone cements containing 7.5 wt% commercial BaSO_4_ (Commercial BaSO_4_ group) and 7.5 wt% porous BaSO_4_@PDA/I-CD/Ag microparticles (BaSO_4_@PDA/I-CD/Ag group) were injected in defect and solidified, respectively.

### 4.5. Mechanical Tests

To evaluate effect of these radiopaque agents on the mechanical property of PMMA bone cements, compression tests were carried out using universal mechanical testing machine (E10000, Instron, Hengyi precision instrument Co., Ltd., Shanghai, China) with three repetitions for each group. In order to investigate the effect of adding an amount of porous BaSO_4_@PDA/I-CD/Ag microparticles in PMMA bone cements on compressive strength, PMMA bone cements containing 0 wt%, 2.5 wt%, 5 wt%, 7.5 wt% and 10 wt% porous BaSO_4_@PDA/I-CD/Ag microparticles were prepared. To compare the effect of different microparticles on compressive strength, PMMA bone cements adding 7.5 wt% commercial BaSO_4_, porous BaSO_4_, porous BaSO_4_@PDA, porous BaSO_4_@PDA/Ag, porous BaSO_4_@PDA/I-CD and porous BaSO_4_@PDA/I-CD/Ag microparticles were then fabricated. The cements were hardened in pillar mold with diameter of 6 mm and height of 12 mm for 1 h and then ejected. The rate for these compression tests were 1 mm min^−1^. Male sheep lumbar vertebrae (L1-L6), decalcified in 10% 10% EDTA Na_2_ (Solarbio, Beijing Solarbio Science & Technology Co., Ltd., Beijing, China) for 20 days, was used to investigate the mechanical property of PMMA bone cement containing 7.5 wt% of porous BaSO_4_, and BaSO_4_@PDA/I-CD/Ag microparticles. The rate for these compression tests were 5 mm min^−1^.

### 4.6. Bacteria Morphology Observation

The antibacterial performance of PMMA bone cements containing porous BaSO_4_, porous BaSO_4_@PDA, porous BaSO_4_@PDA/Ag, porous BaSO_4_@PDA/I-CD and porous BaSO_4_@PDA/I-CD/Ag microparticles were evaluated using *S. aureus* and *E. coli* (supplied by China General Microbiological Culture Collection Center, Beijing, China). Both *S. aureus* and *E. coli* were cultured in Luria-Bertani (LB) medium at 37 °C for 24 h. PMMA bone cements were also detected as control group. The bacteria density was adjusted to be concentration of 10^5^ CFU mL^−1^ in the antibacterial assay. *S. aureus* and *E. coli* were seeded on the bone cements disc with diameter 5.8 mm. After 24 h of incubation, the samples were washed with PBS for three times to eliminate non-adherent bacteria. After fixing in 2.5% glutaraldehyde and dehydrating in graded ethanol/ distilled water mixture (50%, 60%, 70%, 80%, 90% and 100%), the samples were then dried using a Critical Point Dryer (CPD030, LEICA, Wetzlar, Germany) and sputter-coated with gold using an Ion Sputter (SC7620, Quorum Technologies, Lewes, UK). Finally, the morphology of bacteria was observed by SEM.

### 4.7. Antibacterial Efficiency Activity of Cement Containing Particles

The inhibition zone test was employed to evaluate bacterial inhibition activity of particles using a modified disk diffusion method. *S. aureus* and *E. coli* were selected as model bacteria for assay. Briefly, the PMMA bone cement discs (diameter: 10 mm; high: 1mm) containing 7.5 wt% commercial BaSO_4_, BaSO_4_@PDA/Ag and BaSO_4_@PDA/Ag particles were fabricated, 100 μL of fresh bacterial suspension (*S. aureus* and *E. coli*, 10^6^–10^7^ colony forming units per mL) was spread on LB agar plates. Afterward, the PMMA bone cement discs were gently placed on the center of LB agar plates and cultured at 37 °C for 24 h. The size of the zone around disks was measured to evaluate the bacterial inhibition activity of cement containing particles.

### 4.8. Antibacterial Efficiency

The bacterial inhibition rate of PMMA bone cements containing porous BaSO_4_@PDA/Ag or porous BaSO_4_@PDA/I-CD/Ag microparticles compared to PMMA bone cements was studied. Each sample (3 repetitions) was incubated in 10 μL of the bacteria suspension in LB medium at 37 °C for 24 h. Then the samples were gently washed thrice with PBS. Afterwards, the adhered bacteria on each sample were detached into 1 mL of PBS by vortex oscillator for 3 min, and the resulting bacterial suspensions were used to count the viable bacteria adhered on the specimens. The antibacterial rates were calculated based on the following formula:

(Rp) (%) = (B−A)/B × 100%

in which A represents the average number of viable bacteria attached on the PMMA bone cements containing porous BaSO_4_@PDA/Ag or porous BaSO_4_@PDA/I-CD/Ag microparticles, and B represents the number of bacteria on PMMA bone cements.

### 4.9. Cytotoxicity

The cytotoxicity is evaluated by culturing MC3T3-E1 cells using the extracts of PPMMA bone cements containing 7.5 wt% of commercial BaSO_4_, porous BaSO_4_, porous BaSO_4_@PDA, porous BaSO_4_@PDA/Ag, porous BaSO_4_@PDA/I-CD and porous BaSO_4_@PDA/I-CD/Ag particles. The amount of culture medium was added according to the standard of 0.2 mL per gram bone cement. All the bone cements were sterilized for cell culture. The cell growth using the normal culture medium (TCPs group) and the extract of pure PMMA bone cements (PMMA group) were also determined. All the cements were immersed into a MEM alpha modification medium for 24 h, and filtered by 0.22 μm sterile filter head. MC3T3-E1 cells were cultured in 96-well plate (5 × 10^3^ cells/well) with 300 μL normal medium or extracts in a 37 °C incubator with 5% CO_2_. The normal culture medium was prepared by adding 10% (*v*/*v*) FBS and 1% (*v*/*v*) penicillin/streptomycin (Gibco, ThermoFisher Scientific, CA, USA) in MEM alpha modification medium. After culturing of 1, 2 and 3 days, fresh 100 μL MEM alpha modification medium (Hyclone, UT, USA) and 10 μL CCK-8 agent (Dojindo, Kumamoto, Japan) were added and incubated for 2 h in a 37 °C incubator with 5% CO_2_. The absorbance at a wavelength of 450 nm was detected.

### 4.10. The Morphology of MC3T3-E1 Cells on the Surface of Bone Cements

The MC3T3-E1 cells were seeded on the surface of disc of bone cements (diameter: 10 mm, height: 2 mm) in a 24-well plate. For this experiment, pure PMMA bone cement and PMMA bone cements containing commercial BaSO_4_, porous BaSO_4_, porous BaSO_4_@PDA, porous BaSO_4_@PDA/Ag, porous BaSO_4_@PDA/I-CD and porous BaSO_4_@PDA/I-CD/Ag particles were prepared. After culturing for 48 h, the samples were washed three times with PBS, fixed with 4% paraformaldehyde for 40 min, and washed three times with deionized water afterwards. The samples were then dehydrated with gradient ethanol (10%, 30%, 50%, 70%, 70%, 85%, 85%, 90%, 100% and 100%) and dried using a critical point drier. Before SEM observation, all the samples were treated by platinum sputtering for 45 s.

### 4.11. Statistical Analysis

All experiments were performed in triplicate unless otherwise specified. The data were expressed as the means ± standard deviations. Statistical analysis was performed using one-way analysis of variance (ANOVA) followed by Tukey’s multiple comparison test using SPSS software. Differences at *p* < 0.05 were considered statistically significant.

## 5. Conclusions

In this study, we have successfully modified porous BaSO_4_ microparticles with PDA coating containing I-CD and Ag. The fabricated porous BaSO_4_@PDA/I-CD/Ag microparticles with multifunctional coating functioned well for protein loading and delivery as a result of porous structure and the presence of PDA coating and CD on their surface. The presence of I and Ag elements in the coating of BaSO_4_@PDA/I-CD/Ag microparticles led to enhanced X-ray absorbance efficiency. The mechanical property of PMMA bone cements adding porous BaSO_4_@PDA/I-CD/Ag microparticles still meets requirement. In addition, PMMA bone cements containing 7.5 wt% porous BaSO_4_@PDA/I-CD/Ag microparticles show excellent antibacterial property due to the existence of Ag in the coating, and meanwhile possess decent biocompatibility. However, the properties, including cytocompatibility, radiopacity property and antibacterial performance, of bone cements with higher amount of addition of particles should be studied in further study. The in vivo biocompatibility or bone regeneration of PMMA bone cements containing these particles will also be studied in further study. Given the advantage of satisfactory drug delivery capacity, biocompatibility and antibacterial property, this multifunctional coating may also be used for modifying radiopaque agents and other implants.

## Supplementary Materials

Supplementary materials can be found at https://www.mdpi.com/1422-0067/20/9/2055/s1.

## Abbreviations

PMMA	Poly(methyl methacrylate)
I-CD	Hexakis-(6-iodo-6-deoxy)-alpha-cyclodextrin
Ag	Silver
PDA	Polydopamine
BaSO_4_	Barium sulfate
ZrO_2_	Zirconium dioxide
BaCl_2_	Barium chloride
(NH_4_)_2_SO_4_	Ammonium sulfate
PVA	Polyvinyl alcohol
PEEK	Poly(ether ether ketone)
*S. aureus*	*Staphylococcus aureus*
*E. coli*	*Escherichia Coli*
CD	Cyclodextrin
CT	Computed tomography
SEM	Scanning electron microscope
EDS	Energy-dispersive X-ray spectroscope
CCK-8	Cell Counting Kit-8
CuSO_4_	Copper sulfate
BPO	Benzoyl peroxide
MMA	Methyl methacrylate
DMPT	*N*, *N*-dimethyl-p-toluidine
LB	Luria-Bertani
DMF	*N*, *N*-dimethylformamide
H_2_O_2_	Hydrogen peroxide
PBS	Phosphate buffer saline

## References

[1] He Z., Zhai Q., Hu M., Cao C., Wang J., Yang H., Li B. (2015). Bone cements for percutaneous vertebroplasty and balloon kyphoplasty: Current status and future developments. J. Orthop. Transl..

[2] Hernandez L., Fernandez M., Collia F., Gurruchaga M., Goni I. (2006). Preparation of acrylic bone cements for vertebroplasty with bismuth salicylate as radiopaque agent. Biomaterials.

[3] Lissarrague M.H., Fascio M.L., Goyanes S., D’Accorso N.B. (2016). Improving bone cement toughness and contrast agent confinement by using acrylic branched polymers. Mater. Sci. Eng. C Mater. Biol. Appl..

[4] Benzina A., Kruft M.A., van der Veen F.H., Bar F.H., Blezer R., Lindhout T., Koole L.H. (1996). A versatile three-iodine molecular building block leading to new radiopaque polymeric biomaterials. J. Biomed. Mater. Res..

[5] Deb S., Abdulghani S., Behiri J.C. (2002). Radiopacity in bone cements using an organo-bismuth compound. Biomaterials.

[6] Vazquez B., Ginebra M.P., Gil F.J., Planell J.A., Lopez Bravo A., San Roman J. (1999). Radiopaque acrylic cements prepared with a new acrylic derivative of iodo-quinoline. Biomaterials.

[7] Carrodeguas R.G., Lasa B.V., Del Barrio J.S. (2004). Injectable acrylic bone cements for vertebroplasty with improved properties. J. Biomed. Mater. Res. B Appl. Biomater..

[8] Lewis G. (2006). Injectable bone cements for use in vertebroplasty and kyphoplasty: State-of-the-art review. J. Biomed. Mater. Res. B Appl. Biomater..

[9] Ricker A., Liu-Snyder P., Webster T.J. (2008). The influence of nano MgO and BaSO_4_ particle size additives on properties of PMMA bone cement. Int. J. Nanomed..

[10] Lele B., Xiangjun L., Zhenwei W., Jun L. (2018). Fabrication and characterazation of functionalized zirconia microparticles and zirconia-containing bone cement. Mater. Res. Express.

[11] Nandakumar N., Kurian P. (2012). Chemosynthesis of monodispersed porous BaSO_4_ nano powder by polymeric template process and its characterisation. Powder Technol..

[12] Niemann B., Veit P., Sundmacher K. (2008). Nanoparticle precipitation in reverse microemulsions: Particle formation dynamics and tailoring of particle size distributions. Langmuir.

[13] Vallo C.I. (2000). Influence of filler content on static properties of glass-reinforced bone cement. J. Biomed. Mater. Res..

[14] Molino L.N., Topoleski L.D. (1996). Effect of BaSO_4_ on the fatigue crack propagation rate of PMMA bone cement. J. Biomed. Mater. Res..

[15] Ginebra M.P., Albuixech L., Fernandez-Barragan E., Aparicio C., Gil F.J., San R.J., Vazquez B., Planell J.A. (2002). Mechanical performance of acrylic bone cements containing different radiopacifying agents. Biomaterials.

[16] Chen Q.D., Shen X.H. (2010). Formation of Mesoporous BaSO_4_ Microspheres with a Larger Pore Size via Ostwald Ripening at Room Temperature. Cryst. Growth Des..

[17] Rudigier J., Draenert K., Grünert A., Ritter G., Krieg H. (1976). Biological effect of bariumsulfate as contrast material in bone cement. An animal study on rabbit femora (author’s transl). Arch. Orthop. Unfallchir..

[18] Lee H., Dellatore S.M., Miller W.M., Messersmith P.B. (2007). Mussel-inspired surface chemistry for multifunctional coatings. Science.

[19] Lee H., Rho J., Messersmith P.B. (2009). Facile conjugation of biomolecules onto surfaces via mussel adhesive protein inspired coatings. Adv. Mater..

[20] Xu X., Wang L., Luo Z., Ni Y., Sun H., Gao X., Li Y., Zhang S., Li Y., Wei S. (2017). Facile and versatile strategy for construction of anti-inflammatory and antibacterial surfaces with polydopamine-mediated liposomes releasing dexamethasone and minocycline for potential implant applications. ACS Appl. Mater. Inter..

[21] Li R., Chen J., Cesario T.C., Wang X., Yuan J.S., Rentzepis P.M. (2016). Synergistic reaction of silver nitrate, silver nanoparticles, and methylene blue against bacteria. Proc. Natl. Acad. Sci. USA.

[22] Quang Huy T., Van Quy N., Anh-Tuan L. (2013). Silver nanoparticles: Synthesis, properties, toxicology, applications and perspectives. Adv. Nat. Sci. Nanosci..

[23] Miola M., Fucale G., Maina G., Verne E. (2015). Antibacterial and bioactive composite bone cements containing surface silver-doped glass particles. Biomed. Mater..

[24] Gao C., Wang Y., Han F., Yuan Z., Li Q., Shi C., Cao W., Zhou P., Xing X., Li B. (2017). Antibacterial activity and osseointegration of silver-coated poly(ether ether ketone) prepared using the polydopamine-assisted deposition technique. J. Mater. Chem. B.

[25] Cyphert E.L., Learn G.D., Hurley S.K., Lu C.-y., Recum H.A. (2018). An additive to PMMA bone cement enables postimplantation drug refilling, broadens range of compatible antibiotics, and prolongs antimicrobial therapy. Adv. Helthc. Mater..

[26] Feng Q., Wei K., Lin S., Xu Z., Sun Y., Shi P., Li G., Bian L. (2016). Mechanically resilient, injectable, and bioadhesive supramolecular gelatin hydrogels crosslinked by weak host-guest interactions assist cell infiltration and in situ tissue regeneration. Biomaterials.

[27] Rastegari B., Karbalaei-Heidari H.R., Zeinali S., Sheardown H. (2017). The enzyme-sensitive release of prodigiosin grafted β-cyclodextrin and chitosan magnetic nanoparticles as an anticancer drug delivery system: Synthesis, characterization and cytotoxicity studies. Colloids Surf. B Biointerfaces.

[28] Rivera-Delgado E., Djuhadi A., Danda C., Kenyon J., Maia J., Caplan A.I., von Recum H.A. (2018). Injectable liquid polymers extend the delivery of corticosteroids for the treatment of osteoarthritis. J. Control. Release.

[29] Serno T., Geidobler R., Winter G. (2011). Protein stabilization by cyclodextrins in the liquid and dried state. Adv. Drug Deliver. Rev..

[30] van Hooy-Corstjens C.S., Govaert L.E., Spoelstra A.B., Bulstra S.K., Wetzels G.M., Koole L.H. (2004). Mechanical behaviour of a new acrylic radiopaque iodine-containing bone cement. Biomaterials.

[31] Letchmanan K., Shen S.C., Ng W.K., Kingshuk P., Shi Z., Wang W., Tan R.B.H. (2017). Mechanical properties and antibiotic release characteristics of poly(methyl methacrylate)-based bone cement formulated with mesoporous silica nanoparticles. J. Mech. Behav. Biomed..

[32] Fang C., Hua F., Cong Y., Fu J., Cheng Y.-J. (2013). Controlled in situ synthesis of surface functionalized BaSO_4_ nanoparticles for improved bone cement reinforcement. J. Mater. Chem. B.

[33] Zhai Q., Han F., He Z., Shi C., Zhou P., Zhu C., Guo Q., Zhu X., Yang H., Li B. (2018). The “magnesium sacrifice” strategy enables PMMA bone cement partial biodegradability and osseointegration potential. Int. J. Mol. Sci..

