# Supplementary material for: Multifunctional Coating to Simultaneously Encapsulate Drug and Prevent Infection of Radiopaque Agent
Source: Int J Mol Sci. 2019 Apr 26;20(9):2055. doi: 10.3390/ijms20092055 (PMC6539451; doi:10.3390/ijms20092055)
Supplement: Supplementary file 1 [file ijms-20-02055-s001.pdf]

International Journal of
*Molecular Sciences*

# Supplementary Materials

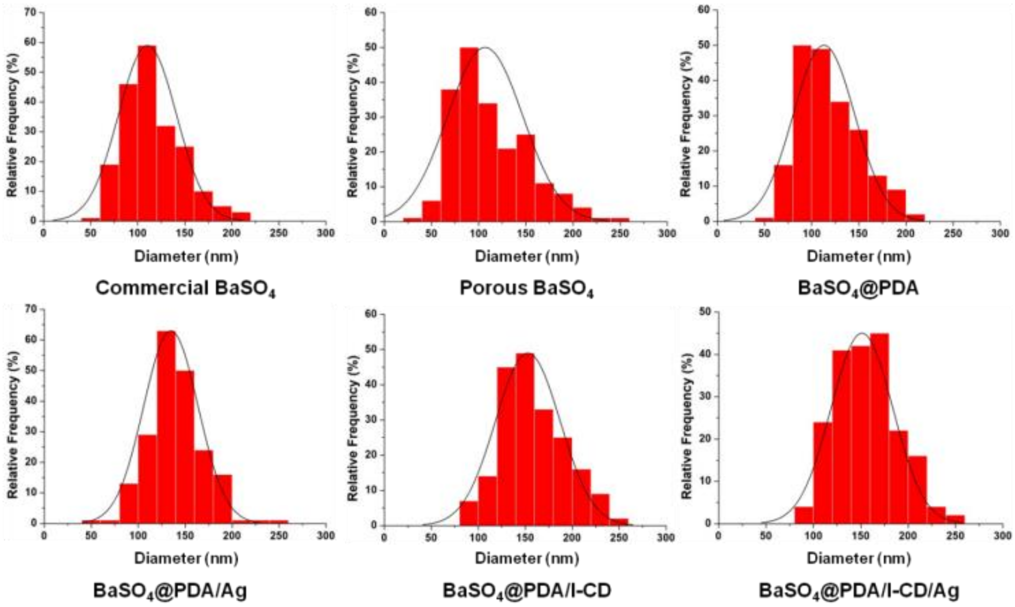


**Figure S1.** The diameter distribution of different microparticles.

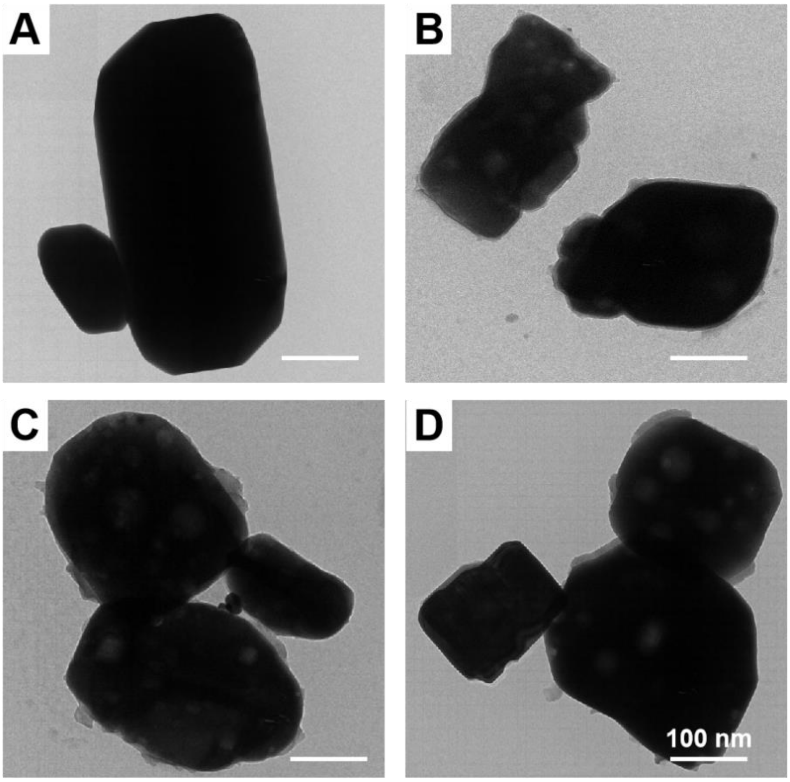


**Figure S2.** TEM images of (A) $BaSO_4$; (B) porous $BaSO_4$@PDA; (C) porous $BaSO_4$@PDA/Ag and (D) porous $BaSO_4$@PDA/I-CD/Ag microparticles.

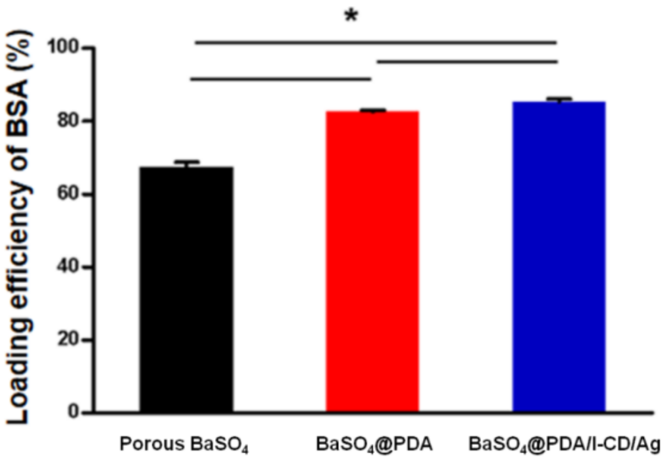


**Figure S3.** The loading efficiency of BSA in porous $BaSO_4$, $BaSO_4$@PDA and $BaSO_4$@PDA/I-CD/Ag particles. *$p < 0.05$.

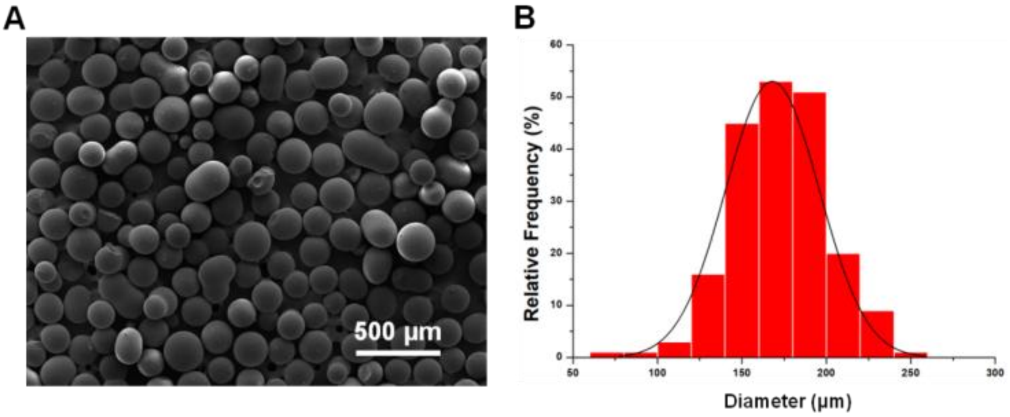


**Figure S4.** (**A**) SEM images of the PMMA for preparing bone cement. (**B**) The diameter distribution of PMMA particles.

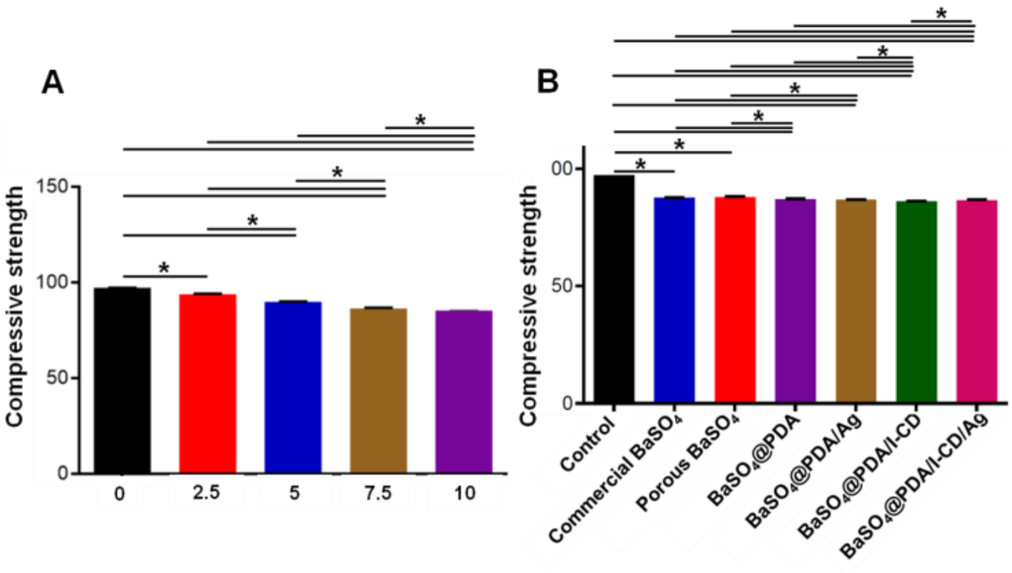

**Figure S5.** (**A**) Compressive strength of PMMA bone cements containing 0, 2.5 wt%, 5 wt%, 7.5 wt% and 10 wt% porous $BaSO_4$@PDA/I-CD/Ag microparticles. (**B**) Compressive strength of PMMA bone cement (Control) and PMMA bone cements adding 7.5 wt% commercial $BaSO_4$, porous $BaSO_4$, $BaSO_4$@PDA, $BaSO_4$@PDA/Ag, $BaSO_4$@PDA/I-CD and $BaSO_4$@PDA/I-CD/Ag microparticles. *$p < 0.05$.

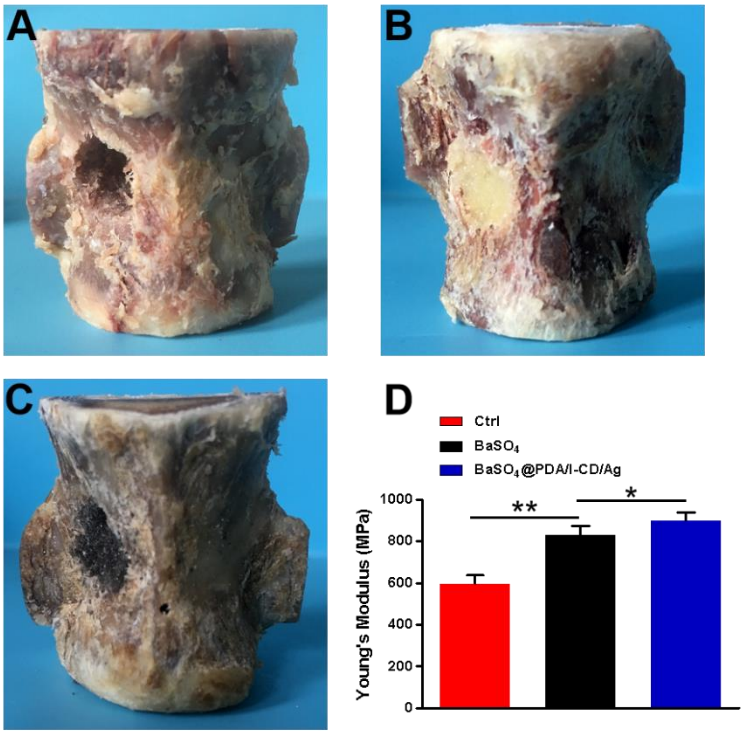


**Figure S6.** Images of sheep vertebral with defect as Ctrl group (**A**), and defects filled with PMMA bone cement containing commercial $BaSO_4$ particles (**B**) and $BaSO_4$@PDA/I-CD/Ag particles (**C**). And Young's modulus of different groups (**D**). *$p < 0.05$.
